# Supplementary material for: Patterns of Intron Gain and Loss in Fungi
Source: PLoS Biol. 2004 Nov 30;2(12):e422. doi: 10.1371/journal.pbio.0020422 (PMC532390; doi:10.1371/journal.pbio.0020422)
Supplement: Table S1 — Also available at http://genes.mit.edu/NielsenEtAl/. (4.3 MB ZIP). [file pbio.0020422.st001.zip › NielsenEtAl/html/754.html]

AN8676.1.NCU07430.1.MG02773.1.FG08696.1


```
 CLUSTAL W (1.82) Multiple Sequence Alignments - Introns Inserted


Sequence 1: NCU07430.1	224 aa
Sequence 2: FG08696.1	224 aa
Sequence 3: MG02773.1	224 aa
Sequence 4: AN8676.1	224 aa
Alignment Length: 236 aa
Number Identitical Residues: 114 aa
Alignment Score (without introns) 4714


MG02773.1 	MAEITDQHDQT-SPTELEDAHSVGNGNSAAQENRGIKRARQSAPDDDDDDDEKGGRERRK
NCU07430.1	MADITDQHEQT-SPTELDDAHAVGNGNVNNNEPRGVKRARPATADDDDDDDDKGGRERRK
FG08696.1 	MADITDQHDQT-SPTELDEQN-VGNG--NATESRGIKRQRPSAGDDDDDDDEKGSRERRK
AN8676.1  	MADITAVGEENPSPTQDDLQQAAGNG---ATDNRGTKRNR-MSADDDDDDEDKTGRERRK
          	**:**   ::..***: :  ::.***     : ** ** *  : ******::* .*****

MG02773.1 	IEIKFINDKSRRHITFSKRKAGIMKK0AYELSVLTGTQVLLLVVSETGLVYTFTTPKLQP
NCU07430.1	IEIKFISDKSRRHITFSKRKAGIMKK0AYELSVLTGTQVLLLVVSETGLVYTFTTPKLQP
FG08696.1 	IEIKFISDKSRRHITFSKRKAGIMKK0AYELSVLTGTQVLLLVVSETGLVYTFTTPKLQP
AN8676.1  	IEIKFIQDKSRRHITFSKRKAGIMKK0AYELSVLTGTQVLLLVVSETGLVYTFTTPKLQP
          	******.******************* *********************************

MG02773.1 	LVTKAEGKNLIQ0ACLNAPEPSAGNENGVDDG--QVDSPEEPASNIPPPQSRA-PGIPSQ
NCU07430.1	LVTKAEGKNLIQ0ACLNAPEPAS-AENGVDDSN-AVDSPEEPSSAHLPPQQGR-PGMP--
FG08696.1 	LVTKSEGKNLIQ0ACLNAPEPTPGNENGVDGGD-QVESPEEPPNQHLPPQGNR-PGMP--
AN8676.1  	LVTKPEGKNLIQ0ACLNAPDPAT-NENGVDAPEVPAETPEDVNHSNVSAQQANIP-----
          	****.******* ******:*:.  *****  .  .::**:      ..*    *     

MG02773.1 	HNPHGMPQYNMPNV-GMDPS---AMAYQSYVQR-GGPQYGMPPQSG~-MPQHSSHQS--
NCU07430.1	PQGHMPPN-YMPAVGAMDAQQAQALAYTNYVQ--QQRGGQYMPQPG~-LQQHAGHQA--
FG08696.1 	QNPHMPPNNYMPNM-PMDPQ--QALAYQSYVQRNQAYGSGIPPQPV1-LVLGGPDSFTR
AN8676.1  	-RPGGMHPAYMTSEQQQQMAYYQNLQQQQQQQQ-QQAGGQYPGMPV~GNRMPTQHQPTA
          	 .        *.     :    . :   .  *            .  .      .. :
```
